# Case Report: Successful Treatment of Dermatomyositis With Telitacicept

**DOI:** 10.1155/carm/2621540

**Published:** 2025-10-13

**Authors:** Xinyu Fu, Miao Yan, Yuan Song, Wei Liang

**Affiliations:** Department of Nephrology, Renmin Hospital of Wuhan University, Wuhan, China

## Abstract

Dermatomyositis is an autoimmune disease. Common treatment with glucocorticoids, immunoglobulins, methotrexate, and rituximab is recommended. Telitacicept is an immunosuppressant that has been approved for the treatment of systemic lupus erythematosus in recent years. Considering the mechanism of action of telitacicept and the pathogenesis of dermatomyositis, it is rational to suppose that telitacicept may be useful in treating dermatomyositis. Here, we describe a case of a 65-year-old male patient with erythema on the face and neck, limb weakness, and edema in both upper and lower limbs. After conventional treatment therapy plus telitacicept, the patient showed significant clinical remission during the maintenance treatment. Methylprednisolone was successfully reduced after injections of telitacicept. After 1 year of follow-up, the patient's clinical symptoms were improved dramatically. This is the first report indicating that telitacicept is effective for dermatomyositis and it deserves attention.

## 1. Introduction

Dermatomyositis (DM) is an autoimmune disease that is clinically heterogeneous. It often results in skin and muscle damage, can lead to multisystem involvement, and can increase the risk of cancer [1]. The focus should be on effectively treating DM after clinical diagnosis. Currently, systemic glucocorticoids, hydroxychloroquine, mycophenolate mofetil, methotrexate, azathioprine, and cyclophosphamide are the conventional first-line drugs recommended for the treatment of DM [2]. If the initial drug treatment is ineffective, second-line and third-line treatments such as rituximab, immunoglobulin, and calcineurin inhibitors are recommended [3]. Telitacicept, a novel immunosuppressant, has been approved for the treatment of active systemic lupus erythematosus (SLE) in China. Clinical trials are also underway to investigate its efficacy in treating other autoimmune diseases, including myasthenia gravis, multiple sclerosis, and Sjogren's syndrome [4]. However, there have been no reports of telitacicept being used in DM. Here, we report a case of DM in which the patient received glucocorticoids and immunoglobulin in combination with telitacicept and eventually recovered well. This case is summarized below for clinical reference.

## 2. Case Presentation

A 65-year-old male patient presented rash around neck and was diagnosed as “dermatitis” in July 2022. Following traditional Chinese medicine and external application treatment, there was no significant improvement. On January 19, 2023, the patient was admitted due to limb weakness, swelling and pain in both upper limbs, edema in both lower limbs, erythema on the neck skin, and oral ulcers. The patient did not report joint pain, dizziness, headache, palpitation, or chest tightness. Since the onset of the disease, the patient's sleep, appetite, and mental status have been normal. However, the physical strength has significantly decreased and the body weight has shown no abnormalities. No past history of hypertension, diabetes, hepatitis B, tuberculosis, blood transfusion, surgical and trauma, and food and drug allergies was reported.

Upon admission, the patient presented with a body temperature of 36.4°C, a pulse rate of 89 beats/min, a respiration rate of 17 times/min, and a blood pressure of 129/76 mmHg. His thorax is symmetrical and his heart rate is regular. Erythema was observed around the neck and a little rash was visible on the face, along with edema and deceased muscle strength at grade 2 level in the upper and lower limbs. There was no edema on the face, no irritation of the jugular vein, no tenderness or rebound pain in the abdomen, and no percussion pain in the bilateral kidney area.

Table 1 shows the results of laboratory examination on admission. The levels of creatine kinase (CK), creatine kinase isoenzymes (CKMB), lactate dehydrogenase (LDH), α-hydroxybutyrate dehydrogenase (α-HBDH), myoglobin, alanine aminotransferase (ALT), and aspartate aminotransferase (AST) were significantly increased. All other laboratory results, including hepatitis B surface antigen (HBsAg), hepatitis B surface antibody (HBsAb), hepatitis B e antigen (HBeAg), hepatitis B e antibody (HBeAb), hepatitis B core antibody (HBcAb), treponemal antibody (TP-Ab), hepatitis C virus antigen (HCVAg), hepatitis C virus antibody (HCV-Ab), and human immunodeficiency virus-antigen/antibody (HIV-Ag/Ab), were within the normal range. None of the following antibodies were detected: anti-neutrophil cytoplasmic antibodies (ANCA), cytoplasmic-antineutrophil cytoplasmic antibody (c-ANCA), perinuclear-antineutrophil cytoplasmatic antibody (p-ANCA), antinuclear antibody (ANA), anti-double-stranded DNA (anti-dsDNA), anti-chromatin, anti-ribosomal P (anti-Ribo-P), anti-SS-A, anti-SS-A/Ro52, anti-SS-A/Ro60, anti-SS-B, anti-centromere protein-B, anti-Smith, anti-ribonucleoprotein (anti-RNP), anti-Smith-RNP, anti-RNP68, anti-RNP-A, anti-scleroderma (anti-Scl-70), and anti-histidyl transfer RNA synthase (anti-Jo-1). Electromyography revealed myogenic lesions in the left upper limb and suspicious lesions in both lower limbs. The results of immunofixation electrophoresis indicted no monoclonal bands in the serum.

Based on the diagnostic criteria for DM, first proposed by Bohan and Peter in 1975, and considering the patient's clinical manifestations and laboratory results, this patient was diagnosed as DM. In 2017, the European League against Rheumatism/American Association of Rheumatology (EULAR/ACR) developed a new classification standard for DM. The standard includes key indicators such as age, skin rash, muscle weakness, elevated CK, LDH, AST, ALT, positive anti-Jo-1 antibody, and positive muscle biopsy [5]. The patient presented with typical symptoms of neck rash, limb edema, and myasthenia. Electromyography revealed myogenic lesion in the left upper limb. Laboratory tests showed significant increases in CK, CKMB, LDH, myoglobin, α-HBDH, AST, and ALT. Although the absence of anti-Jo-1 antibody and muscle biopsy was evident in the patient, the patient's symptoms were in line with a diagnosis of DM.

## 3. Therapeutic Intervention and Outcomes

**Figure 1:**
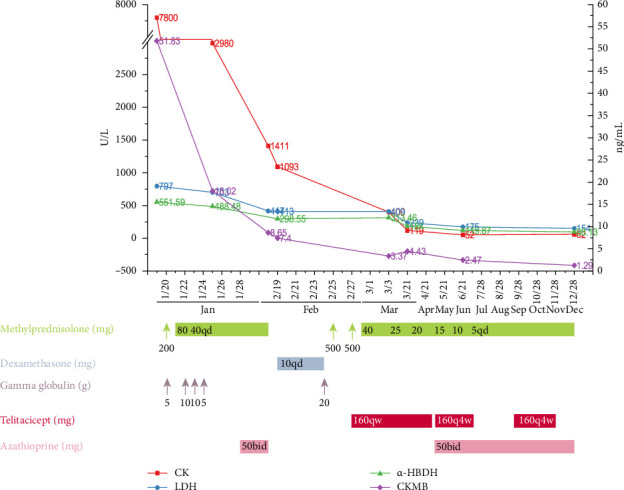
Laboratory examination and timeline diagram of treatment regime and follow-up drug adjustments. LDH: lactate dehydrogenase; α-HBDH: α-hydroxybutyrate dehydrogenase; CK: creatine kinase; CKMB: creatine kinase isoenzymes; qd: every day; qw: once a week; q4w: once every four weeks; bid: twice a day.

In the first hospitalization from Jan 19 to Jan 28 in 2023, a sequential dose of methylprednisolone (200 mg qd for 1 day; 80 mg qd for 2 days; 40 mg qd for 5 days) and gamma globulin (10 g/d for 2 days; 10 g/d for 2 days) was administrated. The serum levels of CK and CKMB decreased significantly (Figure 1). The edema of the limbs had improved, but the erythema of the skin around the neck remained without further deterioration. He was prescribed oral prednisolone (40 mg qd) and azathioprine (50 mg bid) for maintenance treatment.

**Figure 2:**
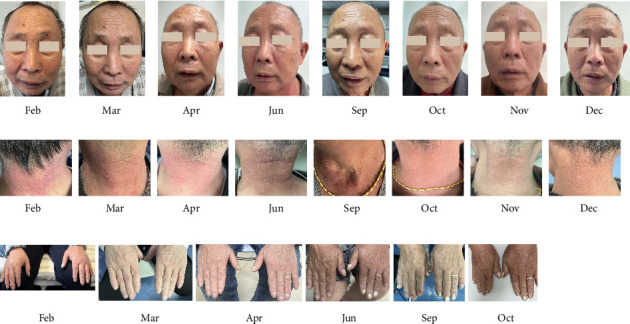
Clinical course. The figure shows the changes of the erythema on the face, erythema in the neck, and edema in the upper limbs, respectively.

On February 18, 2023, the patient was readmitted due to worsening edema and fatigue. A second round of high-dose glucocorticoid was administrated including dexamethasone (10 mg qd for 6 days), methylprednisolone (500 mg qd for 2 days; 40 mg qd for 8 days), and a pulse dose of gamma globulin (20 g/d for 1 day). In order to provide intensive immunosuppressive treatment and to optimize the risk of high dose of methylprednisolone, a medium dose of telitacicept (160 mg qw, Tai'ai, Yantai Rongchang Pharmaceutical, China) was prescribed on February 27, 2023. After 2 doses of telitacicept injection, the general condition including erythema on neck and face as well as limb edema significantly improved (Figure 2). Particularly, the muscle strength in the upper and lower limbs elevated from grade 2 to grade 4. Meanwhile, the elevated levels of CK and CKMB further declined to normal levels, and there was a considerable decrease in LDH and α-HBDH levels (Figure 1). A rapid clinical remission of DM was observed after a short course of telitacicept treatment combined with a regular dosage of methylprednisolone. An intense injection of telitacicept (160 m qw) and medium dose of prednisolone (25 mg qd, tapered 5 mg per month) were prescribed for the second round of maintenance treatment (Figure 1).

Following eight telitacicept injections over the course of 2 months, there was only modest edema on the lower limbs and total recovery of the neck and facial erythema and muscle strength (Figure 2). The levels of CK, CKMB, LDH, and α-HBDH had returned to normal ranges (Figure 1). For the subsequent maintenance treatment, a less frequent injection of telitacicept (160 mg q4w) and a restart of azathioprine (50 mg bid) were administered in accordance with the tapering dose of prednisone (Figure 1).

In the regular follow-up, the patient ceased telitacicept injection in July and August, and the edema on lower limbs and face mildly returned without a flare-up of neck erythema or decreased muscle strength. A less frequent injection of telitacicept (160 mg q4w) was resumed for a period of 2 months (Figure 1). On December 28, 2023, during the follow-up, the patient's general status remained stable with no edema and neck and face erythema and normal muscle strength (Figure 2). The maintenance regimen consisted of 50 mg bid of azathioprine and 5 mg of prednisone taken every day.

The final follow-up was conducted on September 5, 2025, during an outpatient visit. Physical examination revealed no edema or rash on the face, head, neck, or lower extremities. The patient was in good mental and physical condition. Repeat laboratory tests, including complete blood count, liver and kidney function, CK, LDH, α-HBDH, and CKMB, showed no significant abnormalities. Furthermore, the patient reported no recurrence of symptoms or any discomfort since the completion of the previous treatment. Based on these findings, it can be tentatively concluded that the patient with DM has achieved complete clinical remission following comprehensive treatment including telitacicept. During the follow-up, no infection episode or and glucocorticoid-related complication happened.

## 4. Discussion

DM is most usually treated with systemic corticosteroids, immunosuppressants, antimalarial drugs, and intravenous immune globulin. The efficacy of these medications has been established [6]. Corticosteroids are commonly used as the primary treatment for DM. However, the long-term use may have negative effects including adrenal suppression, osteoporosis, reduced immune function, ischemia, and necrosis. Furthermore, intravenous immunoglobulin is typically employed as a short-term treatment option [7]. Minimizing side effects and consequences while treating DM effectively is essential. Second- and third-line medications including rituximab and calcineurin inhibitors are advised in treatment of DM [3]. However, no high-quality trials have demonstrated the effectiveness of the second-line immunosuppressive reagents in DM. Telitacicept is composed of transmembrane activator and calcium regulator and cyclophilin ligand interactor (TACI), which can fuse to the Fc part of human immunoglobulin G (IgG) [8]. It inhibits the differentiation and maturation of B cells and the development and survival of plasma cells by blocking the actions of two signaling molecules—B-cell activating factor (BAFF) and proliferation-inducing ligand (APRIL) [4]. BAFF levels were found to be elevated in patients with DM and decreased following treatment. Additionally, the coexpression of BAFF with CD4^+^ and CD19^+^ B cells in damaged muscles was increased [9]. It is suggested that telitacicept, a medication that targets BAFF and APRIL, may be useful for treating DM. Consequently, we used telitacicept with the patient's fully informed consent and had a successful curative outcome. To our knowledge, this is the first instance of telitacicept being used for DM.

Examining this patient revealed that, following an initial course of treatment consisting of methylprednisolone pulse dose and an intravenous immunoglobulin, the patient's edema and myopathy index had recovered partially. In the first round of maintenance treatment, a full dose of oral methylprednisolone (40 mg qd) and azathioprine (50 mg bid) was less effective in keeping the patient's condition stable, as seen by persistent edema, neck erythema, and decreased muscle strength. After administering a second round of methylprednisolone and intravenous immunoglobulin pulse treatment, a test of telitacicept injection at medium dose in regular frequency (160 mg qw) was recommended. It is interesting to note that after receiving two telitacicept injections in less than 2 weeks, the disorders causing neck erythema, limb edema, and muscular strength grade were considerably improved. In addition, following two rounds of pulse treatment with methylprednisolone and intravenous immunoglobulin, the elevated levels of index of myopathy further decreased, suggesting the potential role of telitacicept during the induction phase of DM treatment.

Following two months of intense telitacicept injection (160 mg qw) and even two months of less intense telitacicept injection (160 mg q4w), the overall condition continued to improve and remained stable. It is noteworthy that a modest case of lower leg edema resulted from the early cessation of telitacicept injection. Conversely, a return to a lower frequency of telitacicept injection (160 mg q4w) reduced the recurrence of edema until the final follow-up, suggesting a beneficial role for telitacicept injection at a lower frequency injection during the maintenance phase of DM treatment.

Of note, the oral glucocorticoid initiated at medium dose of prednisone at 25 mg per day and tapered 5 mg per month in the second round of maintenance treatment, which significantly reduced the accumulative dose of glucocorticoid. These may explain the well-tolerated glucocorticoid tapering and less frequency of episodes of glucocorticoid-related complication occurred during the follow-up. Furthermore, during the tapering off of glucocorticoid and telitacicept during the maintenance period of DM treatment, a medium dose of azathioprine (50 mg bid) was represcribed in the second round of maintenance treatment. This seemed to be better tolerated and more successful than the first round of maintenance treatment after the first round of methylprednisolone pulse treatment.

Based on the therapeutic outcomes observed in this patient, a preliminary assessment of the safety and efficacy of telitacicept in the treatment of DM can be made. However, with regard to adverse effects, the present single case report does not allow for definitive conclusions regarding the side effects of telitacicept in this context. Further investigation through larger cohort studies and well-designed clinical trials is warranted to comprehensively evaluate the potential adverse reactions and long-term safety of telitacicept in patients with DM. The limitations of this study include the following. (1) The therapeutic efficacy of telitacicept may be compromised due to low level of patient compliance. (2) Due to the lack of previous evidence for the treatment of DM with telitacicept, further observation and confirmation of treatment regimen for DM are necessarily needed. (3) The proper options of immunosuppressive agents need to be further confirmed.

## 5. Conclusions

In summary, we presented a case study of telitacicept-treated DM that was successful during the induction and maintenance phases, and we came to the first-ever conclusion that telitacicept may be a significant therapeutic option for DM. To verify this, nevertheless, additional research and clinical trials are required.

## Figures and Tables

**Table 1:** Results of laboratory tests at admission.

Laboratory values (unit)	Patient result	Reference range
Total protein (g/L)	50.9	65–85
Albumin (g/L)	28.1	40–55
Creatinine (μmol/L)	56	57–111
Urea (mmol/L)	5.63	3.6–9.5
ALT (U/L)	114	9–50
AST (U/L)	434	15–40
ALP (U/L)	67	45–125
Fibrinogen (g/L)	6.42	2–4
D-dimer (mg/L)	1.22	0–0.55
NT-proBNP (pg/mL)	6	0–125
Ultra-TnI (ng/mL)	0.012	≤ 0.04
CK (U/L)	> 7800	50–310
LDH (U/L)	797	120–250
α-HBDH (U/L)	551.59	72–182
CKMB (ng/mL)	51.83	0–5
Myoglobin (μg/L)	> 1000	0–110
Hemoglobin (g/L)	135	130–175
WBC count (10^9^/L)	8.96	3.5–9.5
Platelet count (10^9^/L)	431	125–350
CRP (mg/L)	16.44	0–10
ACR (mg/g)	13.6	0–30
24hU-TP (g/24 h)	0.11	0–0.23
IgG (g/L)	9.38	8.6–17.4
IgM (g/L)	0.71	0.3–2.2
IgA (g/L)	1.93	1–4.2
IgE (IU/mL)	109	0–100
Complement C3 (g/L)	0.892	0.7–1.4
Complement C4 (g/L)	0.191	0.1–0.4
PLA2R (RU/mL)	2.67	< 20
Proteinase 3 (RU/mL)	< 2	0–20
BJP	—	—

*Note:* CKMB: creatine kinase isoenzymes; ACR: the ratio of urinary albumin and creatinine; ALT: alanine aminotransferase; AST: aspartate aminotransferase; ALP: alkaline phosphatase; Ultra-TnI: ultra-troponin I; LDH: lactate dehydrogenase; α-HBDH: α-hydroxybutyrate dehydrogenase; PLA2R: phospholipase A2 receptor.

Abbreviations: 24hU-TP, 24-hour urinary-total protein; BJP, Bence Jones protein; CK, creatine kinase; CRP, C-reactive protein; NT-pro BNP, N-terminal pro-brain natriuretic peptide; WBC, white blood cell.

## Data Availability

Data sharing is not applicable to this article as no datasets were generated or analyzed during the current study.
